# Medical History from the Earliest Times

**Published:** 1892-12-10

**Authors:** E. T. Withington


### Medical History from the Earliest Times.

By E. T. Withington, M.A., M.B. Oxon.

XXXV.—THE DARKEST AGE—(*continued*).

Beliefs like those described in the preceding chapter were chiefly confined to the ignorant, but there was another doctrine which threatened not only the progress, but the very existence of medicine, and which was taught by great theologians. If prayer and saintly intercession will heal the sick, is it not an impious want of faith to employ earthly remedies at the same time? Many distinguished rulers of the Church declared that it was. Thus St. Gregory of Tours rebukes Leonastes the Archdeacon because, while trying to cure a disease of his eyes by fasting and prayer at the shrine of St. Martin, he had also called in the aid of a physician. What else could he expect from such an impious proceeding but complete blindness, which actually happened to him? The physician was a Jew, which made the sin worse, as St. Gregory proves from 2 Cor. vi. 14-17, and he concludes with the moral, "Wherefore let this case teach every Christian not to seek earthly aid when he has merited the assistance of heaven." The Bishop would probably have used severer language had he not himself been conscious of a similar error, for once, being troubled with headache, he had not only touched the painful spot with the curtain from before the tomb of St. Martin, but had afterwards been bled by a surgeon, and the thought occurred to him, "as I believe by a suggestion of the enemy," that he might have been cured thus without St. Martin's intercession. He harboured this profane imagination, and was at once punished by a second headache worse than the first, from which he was only relieved by prolonged penitential prayer at the shrine of his predecessor.

At a much later period, we find St. Bernard of Clairvaux strictly forbidding his sick monks to have anything to do with physic or physicians. Let them think of the words of the Apostle (2 Cor. xii. 9), and hesitate to use earthly remedies at the risk of their eternal salvation. The occasional use of herbs from the monastery garden may, indeed, be tolerated; "but to buy drugs, to consult physicians, to take medicines, befits not religion (*religioni indecens est*), and is contrary to purity, and especially is it contrary to the purity and honour of our order, for after all these things do the Gentiles seek, but we know that they who live in the flesh cannot please God." Yet even Cistercians sometimes took medicines as we learn by the following letter from the saint to one of his abbots. "Know that Brother G ——, since he came from Crista, where he took medicines, has had no indulgence in diet, neither has he been excused the usual vigils. Should he, therefore, attempt to obtain these privileges with you, consent not, for you may be certain that it is not his body but his spirit that is affected. Farewell." Had Brother G —— avoided medicines he might, perhaps, have been treated with less suspicion, if not with more indulgence.

The idea that the highest piety is incompatible with the use of medicine comes out also in the legends of the saints, and we may find a striking instance of it in the story of St. Agatha. According to her biographer, Simeon Metaphrastes, St. Peter appeared to her in prison under the guise of an aged leech and offered to heal her wounds. But the saint repulsed him with horror. Never in her life, she declared, had she used other means than prayer for the cure of diseases, was she likely to fail now? And the apostle was finally obliged not only to reveal his name and mission, but to accomplish the cure without the use of physical remedies.

But most were less scrupulous, and one of the pleasantest pictures in an age presenting little of that character is the peaceful monk, gathering his herbal simples and brewing therefrom decoctions which were, doubtless, comforting to the minds, and sometimes also to the bodies of his neighbours. A specially attractive figure is that of Walafrid with the Squint (Strabus), monk of St. Gallen and Abbot of Reichenau, A.D. 850, who has left us twenty-three little poems, in excellent Latin, on the favourite herbs in his monastery garden. For outward griefs he considers nothing equal to oil of roses, the virtues of which are innumerable—

> "Quod quam saepe fiat mortalibus utile curis,
> Nec meminisse potest hominum nec dicere quisquam."

but in internal affections he gives the palm to a decoction of horehound, which is equally valuable in respiratory and digestive disorders, and will even counteract the poisons of malicious stepmothers!

To the long list of Arab patrons of science during this period we can only oppose two names, those of Alfred the Great, and Charlemagne. The former had, perhaps, something to do with the origin of the curious "Anglo-Saxon Leechdoms," which the reader will find translated in the Master of the Roll's series. They comprise imperfect translations from the works of Dioscorides, Apuleius, and Sextus Placitus, together with a few original productions, which, however, display the wit rather than the wisdom of our ancestors. Here are two examples: "Against a woman's chatter; take at night, fasting, a root of radish; that day the chatter cannot harm thee." "In case a man be a lunatic; take skin of a meer-swine or porpoise; work it into a whip; swinge the man therewith; soon he will be well; Amen." Charlemagne seems to have done less for medicine than for other sciences, for, as his biographer Einhard tells us, "he especially hated physicians because they tried to make him take his meat boiled instead of roast; wherefore he would have none of them, but treated himself when ill, which rarely happened till the last four years of his life." In 806, however, he ordained that children (infantes) should be sent to learn medicine, and it is perhaps the practical results of this edict which are quaintly and prophetically described in the following lines by his English "Minister of Education" Alcuin—

> Accurrunt medici mox Hippocratica tecta,

> Hic venas fundit, herbas hic miscet in ollâ

> Ille coquit pultes, alter sed pocula praefert.

> "Soon hasten to halls Hippocratic in crowds the medical students;
> One is opening a vein, another is stirring a mixture,
> Here one is cooking the gruel, there one is dosing a patient."

But the passage itself is so obscure, and the great Emperor's institutions so entirely vanished in the disorders which followed his death, that it is impossible to decide whether he intended to establish medical schools, or by making some knowledge of the science universal, to entirely abolish a profession which held such strange views on the relative excellence of roast and boiled meats. He seems, however, to have done something for the furtherance of State medicine, for, according to Haeser, he admitted the ecclesiastical ordinance that each parish should care for its poor into his code of laws, declared the hospitals to be State institutions, and had them inspected periodically by special officers (*missi dominici*). Finally, Charlemagne benefited the healing art by commanding that useful plants, especially those of a medicinal character, should be cultivated on all the royal farms.

